# Cancrum Oris

**Published:** 1881-12-20

**Authors:** B. F. Duke

**Affiliations:** Mississippi


### CANCRUM ORIS.

BY B. F. DUKE, M. D., OF MISSISSIPPI.

In 1878 Dr. J. L. Hudson and myself treated two cases of *cancrum oris*, both of which proved fatal.

A slough had formed on the outside of the cheek before we saw them, they having been treated by others for *mercureal stomatitis*. These patients, both children of two years, survived until the entire cheek and lips on that side had sloughed away. Shortly afterwards we saw another case, which had not advanced so far; and this one recovered. Having taken no notes, I cannot recall the treatment in full; but remember that sulphate of copper and other astringent applications were used.

Recently, I have had two other cases, following prostrating attacks of remittent fever, in which the following treatment succeeded to my perfect satisfaction: The gangrenous patches were broken up with a bistoury, and a pencil of sulphate of copper thoroughly applied three times a day afterwards. In addition to this a finely powdered mixture of alum, borax and chlorate of potash, one part each, and four of white sugar, was placed in the mouth directly in contact with the diseased part every two or three hours, and the swollen cheek bathed frequently with spirits of camphor, such as the family had in the house, made with whisky.

The following constitutional treatment was used in both cases—

| | | |
|---|---|---|
| ℞ | Sulph. cinchonidia, | ʒj, |
| | Sulph. magnesia, | ʒij, |
| | Muriatic acid, | f. ʒj, |
| | Glycerine and water, | aa ℥ij |

M. S. A teaspoonful every four hours.

The tincture of opium was afterwards added to restrain diarrhœa, and the salts, which was used for constipation, diminished.

These children were brothers, aged 3 and 6 years. The second was attacked as the first was recovering, each case being of about ten days duration from the time the disease was first noticed. The treatment began on the second day and continued without any appreciable improvement for two days.

Concentrated nourishment was given every three or four hours, even in the absence of any desire for food, which was the case until convalesence was fully established.

